# FUCA2 Is a Prognostic Biomarker and Correlated With an Immunosuppressive Microenvironment in Pan-Cancer

**DOI:** 10.3389/fimmu.2021.758648

**Published:** 2021-10-20

**Authors:** Anyuan Zhong, Ting Chen, Yufei Xing, Xue Pan, Minhua Shi

**Affiliations:** Department of Pulmonary and Critical Care Medicine, The Second Affiliated Hospital of Soochow University, Suzhou, China

**Keywords:** FUCA2, prognosis, tumor associated macrophages, immunosuppressive microenvironment, pan-cancer

## Abstract

**Background:**

The expression of Fucosidase, alpha-L-2 (FUCA2) varies across tumors. However, its role in various tumor types and relationship with the tumor immune microenvironment (TIME) is poorly defined.

**Methods:**

We analyzed profiles of FUCA2 expression using datasets from The Cancer Genome Atlas (TCGA) and Genotype-Tissue Expression (GTEx) databases. Next, gene alteration, clinical characteristics and prognostic values of FUCA2 were elucidated based on TCGA pan-cancer data. This was followed by gene set enrichment analysis by R software. Relationships between FUCA2 expression and immune infiltration and immune-related genes were also evaluated. Moreover, the association of immune cell infiltration with FUCA2 expression was evaluated across three different sources of immune cell infiltration data, namely the TIMER online, ImmuCellAI databases, as well as a published study. In addition, MTT assays was also conducted to validate the oncogene role of FUCA2 in lung cancer cells.

**Results:**

FUCA2 was upregulated in most tumors, and this was significantly associated with poor survival rates. Gene set enrichment analysis uncovered that FUCA2 correlated with immune pathways in different tumor types. FUCA2 expression was positively related to tumor associated macrophages (TAMs), especially M2-like TAMs. Moreover, FUCA2 level showed a positive relationship with most immunosuppression genes, including programmed death-ligand 1 (PD-L1), transforming growth factor beta 1 (TGFB1), and interleukin-10 (IL10) in most cancer types. FUCA2 knockdown inhibited the cell viability in lung cancer cells.

**Conclusions:**

Our study reveals that FUCA2 is a potential oncogene and is indicative biomarker of a worse prognosis in pan-cancer. High FUCA2 expression may contribute to increased infiltration of TAMs and associates with an immunosuppressive microenvironment, providing a potential target for tumor therapy.

## Introduction

Recent advancements and popularization of sequencing technology have led to identification of numerous oncogenes, most of which are biomarkers for diagnosis and prognosis of malignant tumors. Typical tumor oncogenes, such as catenin beta 1(CTNNB1), hypoxia inducible factor 1, alpha subunit (HIF1α), have been identified and found to enhance tumor development (1–4). Previous studies have shown that the tumor microenvironment (TME) disorder, especially tumor immune microenvironment (TIME), is one of the main causes of malignant progression (5). For example, accumulation of immune infiltrating cells in TME was previously implicated in cancer development (6). Previous studies have demonstrated that oncogenes can rebuild tumor microenvironment and promote tumor progression by directly or indirectly affecting immune cells or stromal cells (7). Furthermore, recent years, cancer immunotherapy has shown clear benefits in the survival of cancer patients, especially immune checkpoint inhibitors (ICIs), such as programmed death- 1 (PD-1), programmed death ligand-1 (PD-L1), and cytotoxic T lymphocyte associated antigen 4 (CTLA4) inhibitors (8). However, most patients are still not sensitive to ICIs treatment, which may be related to the lack of biomarkers to guide personalized immune targets. It was generally reported that the efficacy of immunotherapy is associated with TIME (9). Based on this, we sought to identify genes that play key roles in TIME.

To date, only a handful of studies have reported the role played by Fucosidase, alpha-L- 2 (FUCA2), a gene that codes for a secreted non-lysosomal enzyme, in tumor development (10). For example, recent studies revealed this gene plays an important diagnostic role in hepatocellular carcinoma (11). In addition, FUCA2 was found showed diagnostic and therapeutic value in helicobacter pylori-infected gastric cancer (12). Despite these findings, additional research is needed to unravel more roles played by FUCA2 in tumor development and progression.

Herein, a comprehensive analysis of the FUCA2 function was carried out using multi-omics datasets for 33 cancers from the TCGA database, and further explored the link between FUCA2 level and the TIME. Results revealed that the gene has a diagnostic role in pan-cancer. Particularly, overexpression of FUCA2 predicted the immunosuppressive tumor microenvironment, which may induce poor survival rates in patients with tumors. Collectively, the obtained results indicate that FUCA2 may be a potential target for future development of cancer therapies.

## Materials and Methods

### Data Collection

RNA sequence and related clinical data (comprising 11069 samples from 33 types of cancer) were retrieved from TCGA database using UCSC Xena (https://xena.ucsc.edu/). Gene profile data from normal human tissues were retrieved from GTEx (https://commonfund.nih.gov/GTEx), while analysis of Clinical proteomic tumor analysis consortium (CPTAC) data was performed on the UALCAN portal (http://ualcan.path.uab.edu/analysis-prot.html). T-tests was used to detect the differences of expression between tumor and normal tissues; P<0.05 were considered to indicate Statistical analyses were conducted using R software (version 4.0.2). The R package “ggplot2” was used to draw box plots.

### Prognostic Value of FUCA2

Univariate Cox regression (uniCox) and Kaplan-Meier analyses were conducted to explore the influence of FUCA2 on the survival of patients in pan-cancer using R package “survminer” and “survival”. Overall survival (OS), disease-specific survival (DSS), disease-free interval (DFI), and progression-free interval (PFI) were evaluated (P < 0.05 as significant).

### Analysis of Copy Number Alterations and DNA Methylation inFUCA2

Copy number alterations (CNA), mutations and DNA methylation alterations of FUCA2 in pan-cancer patients were determined using the cBioPortal for Cancer Genomics (http://www.cbioportal.org/) platform. Thereafter, Kaplan-Meier survival curves were designed to reveal the link between FUCA2 methylation and clinical outcomes of patients (P < 0.05 as significant).

### Gene Set Enrichment Analysis and Gene Set Variation Analysis

GSEA and GSVA were employed to investigate the potential biological process of FUCA2 in pan-cancer. GSEA was performed using R packages “clusterprofiler” (13). Adjusted P-values < 0.05 were considered statistically significant. GSVA is commonly applied for estimating the variation in pathway and biological process activity in the samples of an expression dataset (14). The Hallmark pathway gene set was downloaded from the Molecular Signatures Database (MSigDB), and the Hallmark pathway scores were obtained for all cancers using the R language “GSVA” package.

### Relationship Between FUCA2 Expression With TME and Immune Cell Infiltration

Three different methods were then employed in the evaluation of FUCA2 level versus immune cell infiltration relationship in pan-cancer. In the first method, the TIMER2 database (http://timer.comp-genomics.org/) was examined to elucidate the association of FUCA2 level with macrophage infiltration. In the second, we downloaded immune cells infiltration data for TCGA pan-cancer patients, based on a previous study (15), which was then subjected to correlation analysis. In the third approach, immune cells infiltration scores for TCGA pan-cancer patients were retrieved from the ImmuCellAI platform (http://bioinfo.life.hust.edu.cn/web/ImmuCellAI/). The data was processed through correlation analysis. The correlation of FUCA2 expression with immune-related genes, including those encoding chemokine receptor proteins, chemokine, immunosuppressive, and major histocompatibility complex (MHC) was also analyzed. Pearson correlation coefficients were calculated and an expression heat map was showed for each type of cancer.

#### Cell Culture

Lung cancer cell lines A549 and NCI-H1299 were purchased from the American Type Culture Collection (ATCC: Manassas, VA, USA). Cell lines were cultured in RPMI-1640 medium (Gibco, China), with 10% fetal bovine serum (Gibco, China) and were grown in an atmosphere of 5% CO2 at 37^◦^C.

#### RNA Extraction and qRT-PCR

According to the manufacturer’s protocol, total RNA were isolated using TRIzol reagent (Pufei, Shanghai, China). The primers sequences used for qRT-PCR were obtained from Applied Biosystems (Ribo, Guangzhou, China) as below (5’-3’): FUCA2 forward - GGAGGGAAGCTGGAATCTC, reverse- CTTTTAGCCAGGACCCCAT. GAPDH forward-GCGTGACATTAAGGAGAAGC, reverse- CCACGTCACACTTCATGATGG. Relative expression levels were calculated according to the 2-∆∆Ct method. Statistical analyses were performed with GraphPad Prism.

#### Lentiviral Transduction

The siRNAs for FUCA2 (NM_032020) were ligated into the GV493 plasmid (GeneChem). The sequence for the control siRNA (NC) was as follows: 5′- TTCTCCGAACGTGTCACGT -3′. The sequences for FUCA2 siRNA were as follows: 5′-TGGAATCTCTGACTATCTT-3′, and 5′-TCTATGAGTTAGTGAACAA-3′. The supernatant was removed 24 h after infection and replaced with D10 medium. qRT-PCR analyses verified that lentiviral transduction downregulated the level of FUCA2 mRNA.

#### Methyl-Thiazolyl-Tetrazolium Assay

Cell viability was detected using MTT assay. The transfected cells were inoculated into 96-well plates with 2×10^3^ cells per well. At 24, 48, 72, 96 and 120 hours, 20 μL MTT solution was added to the medium and cells were incubated for 4 hours. After discarding the medium, 200 μL DMSO was added to the cells and the formazan crystal was dissolved for 15 minutes. The optical density (OD) value at 490 nm was detected by the enzyme-linked immunometric meter (Thermo Fisher Scientific).

## Results

### FUCA2 Is Upregulated in Pan-Cancer Tissues

**Figure 1:**
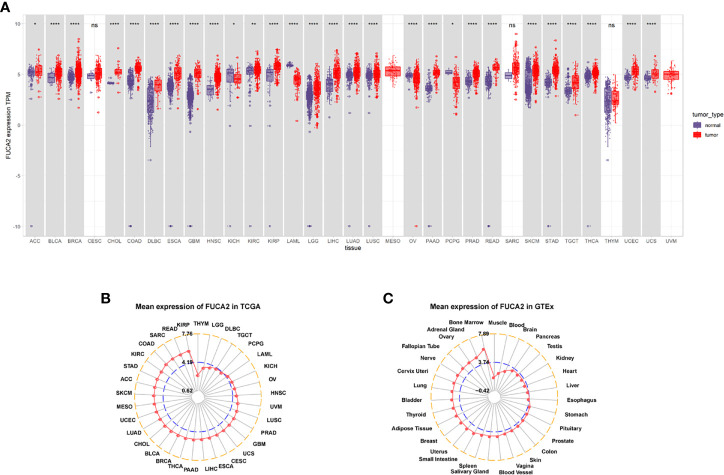
FUCA2 levels varies across tumors. **(A)** Patterns of FUCA2 level between tumor and normal tissues. **(B)** Profiles of FUCA2 expression in 33 cancer types. **(C)** Expression of FUCA2 in normal tissues. *P < 0.05, **P < 0.01, ****P < 0.0001.

**Figure 2:**
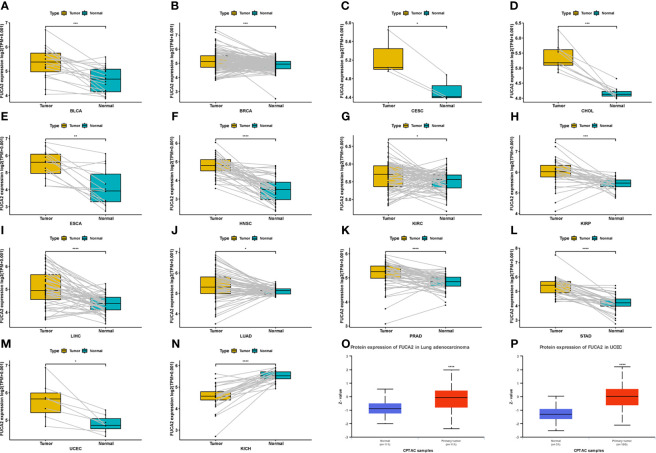
Patterns of FUCA2 levels between tumor and normal tissues. **(A–N)** levels of FUCA2 in paired tumor and normal tissues across different cancers based on a TCGA dataset. **(O, P)** Protein expression level of FUCA2 in LUAD and UCEC. *P < 0.05, **P < 0.01, ***P < 0.001, ****P < 0.0001.

Analysis of FUCA2 mRNA expression in datasets from GTEx and TCGA databases revealed upregulation of this gene in tumor tissues from uterine carcinosarcoma (UCS), uterine corpus endometrial carcinoma (UCEC), thyroid carcinoma (THCA), testicular germ cell tumors (TGCT), skin cutaneous melanoma (SKCM), rectum adenocarcinoma (READ), prostate adenocarcinoma (PRAD), pancreatic adenocarcinoma (PAAD), lung squamous cell carcinoma (LUSC), lung adenocarcinoma (LUAD), liver hepatocellular carcinoma (LIHC), brain lower grade glioma (LGG), kidney renal papillary cell carcinoma (KIRP), kidney renal clear cell carcinoma (KIRC), head and neck squamous cell carcinoma (HNSC), glioblastoma multiforme (GBM), esophageal carcinoma (ESCA), lymphoid neoplasm diffuse large B-cell lymphoma (DLBC), colon adenocarcinoma (COAD), cholangiocarcinoma (CHOL), breast invasive carcinoma (BRCA), bladder urothelial carcinoma (BLCA), adrenocortical carcinoma (ACC), stomach adenocarcinoma (STAD). By contrast, it exhibited low expression in pheochromocytoma and paraganglioma (PCPG), ovarian serous cystadenocarcinoma (OV) acute myeloid leukemia (LAML), acute myeloid leukemia (LAML), and kidney chromophobe (KICH), relative to normal tissues (**Figure 1A**). Profiles of FUCA2 expression across various types of cancers, as well as levels across different tissues are presented, and ranked from low to high, in **Figures 1B, C**. For paired tumors in the TCGA dataset, FUCA2 was upregulated in UCEC, STAD, PRAD, LUAD, LIHC, KIRP, HNSC, ESCA, KIRC, CHOL, CESC, BLCA, and BRCA, relative to adjacent normal tissues (**Figures 2A–M**). However, its expression was downregulated in KICH, relative to adjacent normal tissues (**Figure 2N**). Results of the CPTAC dataset revealed that FUCA2 protein was upregulated in primary tissues of LUAD and UCEC, relative to normal tissues, after analysis on the UALCAN database (**Figures 2O, P**).

### Impact of FUCA2 on Pan-Cancer Prognosis

**Figure 3:**
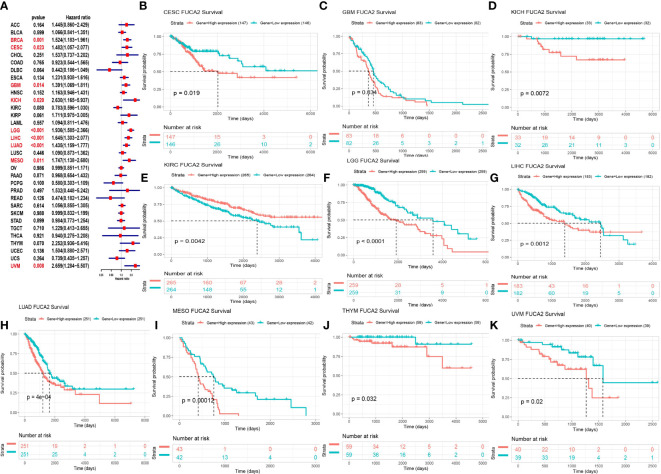
Relationship between FUCA2 level and OS. **(A)** Forest plot showing OS after Cox analysis in pan-cancer. Red color represents significant results (p < 0.05). **(B–K)** Kaplan-Meier curves showing OS in pan-cancer. Only significant results were shown.

We used Cox proportional hazards model to elucidate the relationship of FUCA2 level with overall survival (OS) of patients in TCGA pan-cancer. Results revealed that high FUCA2 level was significantly linked to worse OS in patients with BRCA (p= 0.001), CESC (p=0.023), GBM (p=0.014), KICH (p= 0.020), LGG (p<0.001), LIHC (p<0.001), LUAD (p<0.001), MESO (p = 0.011), and Uveal Melanoma (UVM) (p = 0.008) (**Figure 3A**). Moreover, Kaplan–Meier curves uncovered that high levels of FUCA2 expression were associated with shorter survival times in patients with CESC (p = 0.019), GBM (p = 0.034), KICH (p = 0.0072), LGG (p<0.0001), LIHC (p = 0.0012), LUAD (p = 4e-04), MESO (p = 0.00012), UVM (p = 0.02). In contrast, high FUCA2 expression in KIRC (p = 0.0042), were associated with longer OS times (**Figures 3B–K**).

**Figure 4:**
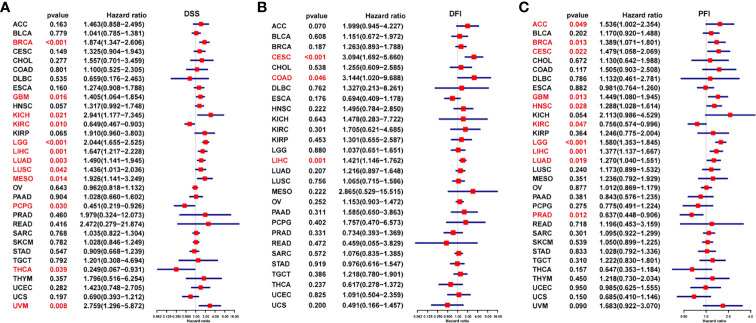
Prognostic value of FUCA2 based on a TCGA dataset in pan-cancer. Forest plots showing results of Univariate Cox Regression analysis for **(A)** DSS, **(B)** DFI, and **(C)** PFI. Red color represents significant results (p < 0.05).

Next, we generated forest plots after performing univariate Cox regression analysis for progression-free interval (PFI), disease-free interval (DFI), and disease-specific survival (DSS). For DSS, low FUCA2 expression was significantly associated with worse clinical outcomes of KIRC (P = 0.010), PCPG (p = 0.030), and THCA (P = 0.039) (**Figure 4A**). However, better DSS rates were observed in patients with low FUCA2 expression in GBM (p = 0.016), KICH (p = 0.021), LGG (p=0.016), LIHC (p =0.001), LUAD (p = 0.003), LUSC (p = 0.042), MESO (p = 0.014) and UVM (p = 0.008). For DFI, high FUCA2 expression was associated with significantly lower DFI in patients with CESC (p<0.001), COAD (p =0.046) and LIHC (p = 0.001) (**Figure 4B**). On the other hand, it was evident that high FUCA2 level correlated with significantly poor PFI in patients with ACC (p= 0.049), BRCA (p= 0.013), CESC (p= 0.022), GBM (p= 0.013), HNSC (p= 0.028), LGG (p < 0.001), LIHC (p = 0.001) and LUAD (p =0.019), while low FUCA2 level was significantly linked to better PFI in patients with KIRC (p = 0.047) and PRAD (p = 0.012) (**Figure 4C**).

### CNA and DNA Methylation of FUCA2 In Pan-Cancer

**Figure 5:**
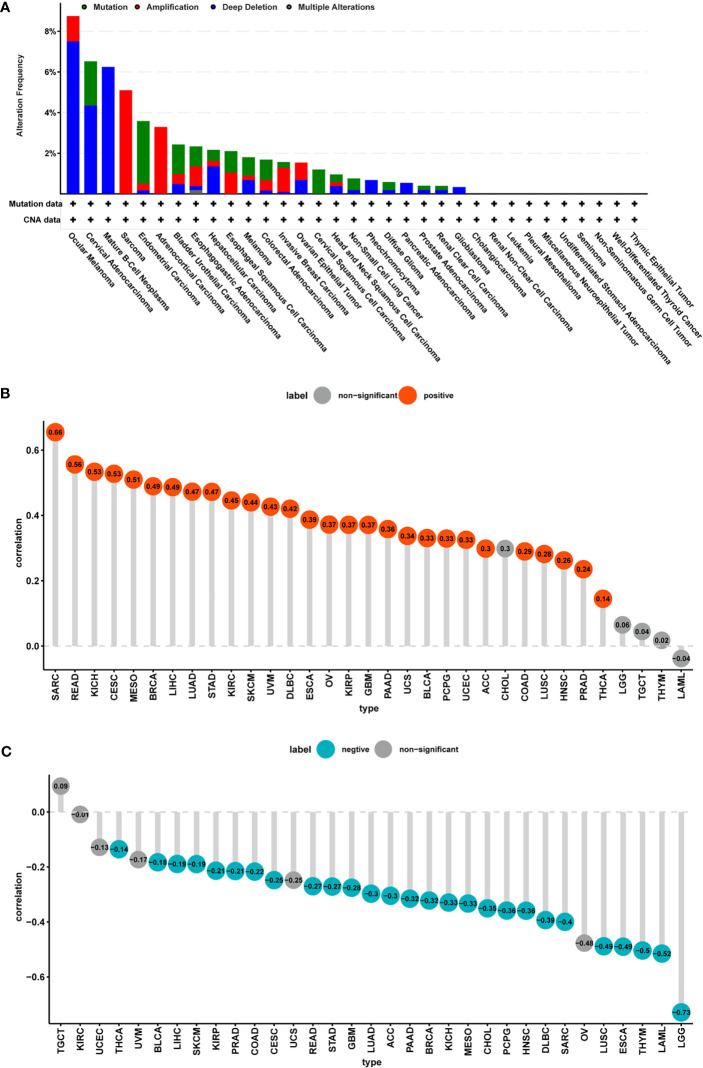
CNA and DNA methylation of FUCA2 in pan-cancer. **(A)** The mutation and CNA status of FUCA2 in TCGA-pan-cancer using cBioportal database. **(B)** Correlation between expression levels of FUCA2 mRNA and DNA copy number. Red color represents significant results (p < 0.05). **(C)** Correlations between mRNA level of FUCA2 and DNA methylation. Blue color represents significant results (p < 0.05).

Analysis of genetic alterations in FUCA2 using cBioPortal revealed that the highest frequency of alteration in this gene (>8%) occurred in patients with ocular melanoma with “deep deletion” as the primary type. The “amplification” type of CNA was the primary type in the sarcoma cases, as evidenced by a ~5% alteration frequency (**Figure 5A**). Thereafter, we analyzed the relationship between gene expression and relative linear copy number values and found that FUCA2 expression was positively correlated with CNA in tumor types, except for CHOL, LGG, TGCT, THYM, and LAML (**Figure 5B**). Furthermore, we used Pearson’s correlation to explore the relationship between the status of promoter DNA methylation with levels of FUCA2 expression (**Figure 5C**). Results showed that DNA methylation had a significant negative correlation withFUCA2 expression in patients with LUAD, ACC, PAAD, BRCA, KICH, MESO, CHOL, PCPG, HNSC, DLBC, SARC, LUSC, ESCA, THYM, LAML and LGG (−1<Pearson r≤−0.3), and in THCA, BLCA, LIHC, SKCM, KIRP, PRAD, COAD, CESC, READ, STAD and GBM (−0.3 < Pearson r < −0.1).

**Figure 6:**
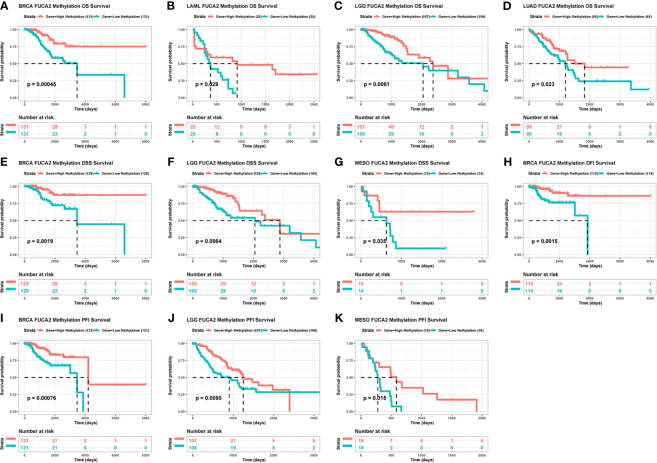
Correlation between FUCA2 methylation and survival of tumor patients. **(A–D)** Correlation between FUCA2 methylation and OS in BRCA, LAML, LGG and LUAD. **(E–G)** Correlation between FUCA2 methylation and DFS in BRCA, LGG and MESO. **(H)** Correlation between FUCA2 methylation and DFI in BRCA. **(I–K)** Correlation between FUCA2 methylation and PFI in BRCA, LGG, and MESO. Only significant results were shown.

We then generated Kaplan–Meier curves to ascertain the prognostic value of FUCA2 promoter methylation in TCGA pan-cancer dataset. Results indicated that the level of FUCA2 methylation was a prognostic factor for OS of patients with BRCA, LAML, LGG and LUAD (**Figures 6A–D**). With regards to DSS, FUCA2 methylation was a prognostic factor for patients with BRCA, LGG and MESO (**Figures 6E–G**). However, the level of FUCA2 methylation was only associated with increased DFI in patients with BRCA (**Figure 6H**), with low FUCA2 methylation level significantly associated with reduced PFI in patients with BRCA, LGG, and MESO (**Figures 6I–K**).

### Analysis of Immune Cell Infiltration

**Figure 7:**
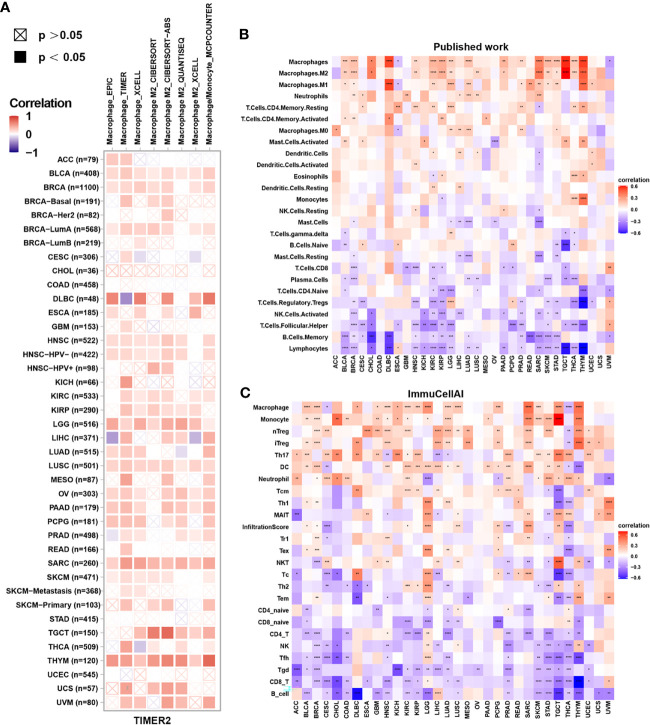
Correlation between FUCA2 expression and levels of tumor infiltration across different immune cells. **(A)** Correlation between FUCA2 expression and macrophages from TIMER2 database. **(B)** Correlation between FUCA2 and different immune cells from published study. **(C)** Correlation between FUCA2 expression and different immune cells from ImmuCellAI database. Red represents positive correlation, blue represents negative correlation, and the darker the color, the stronger the correlation. *P < 0.05, **P < 0.01, ***P < 0.001, ****P < 0.0001.

Tumor-infiltrating immune cells are typically dysfunctional, fail to control tumor growth and may even promote its progression, leading to immune escape. Enrichment of tumor-associated macrophages (TAMs) in the TME is linked to tumor initiation and progression. Therefore, we assessed the correlation between FUCA2 expression and macrophage infiltration using TIMER2. Interestingly, the level of TAM infiltration was significantly positively associated with FUCA2 expression in most tumor types, especially M2-like TAMs (**Figure 7A**).

Next, we assessed the correlations between immune cell infiltration and FUCA2 expression in 33 cancers using a published work and the ImmuCellAI database. A clustering heatmap showed a positive correlation between FUCA2 and TAMs, consistent with the TIMER2 results (**Figures 7B, C**). Additionally, FUCA2 was negatively associated with CD8+ T cells in several cancers (**Figures 7B, C**).

### Relationship Between FUCA2 Expression With Immune-Related Genes

**Figure 8:**
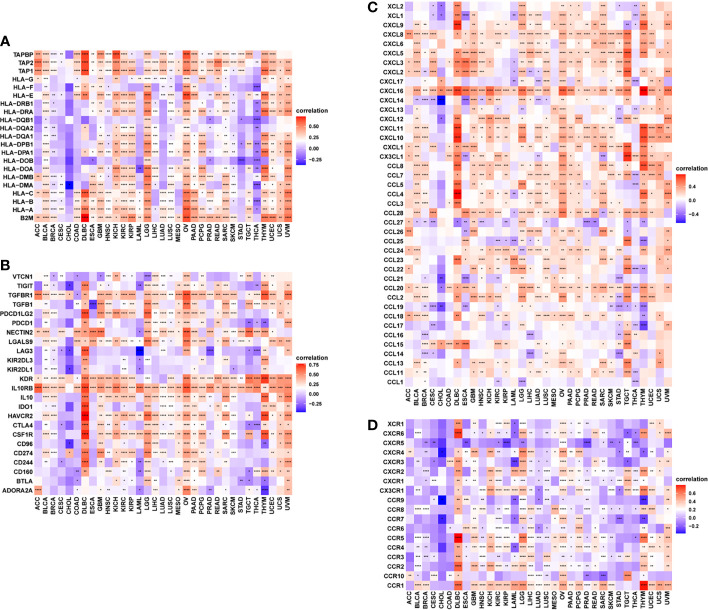
Relationship between FUCA2 expression and that of immune-related genes. **(A)** MHC genes. **(B)** Immunosuppressive genes. **(C)** Chemokines **(D)**. Chemokine receptors. Red represents positive correlation, blue represents negative correlation, and the darker the color, the stronger the correlation. *P < 0.05, **P < 0.01, ***P < 0.001, ****P < 0.0001.

Furthermore, gene co-expression analyses, conducted to explore the relationship between FUCA2 expression and immune-related genes in pan-cancer, revealed that FUCA2 expression was positively correlated with most MHC genes (**Figure 8A**), immunosuppressive genes (**Figure 8B**), chemokines (**Figure 8C**), and chemokine receptors (**Figure 8D**) across most tumor types (**Supplementary Table 1**). In these immunosuppressive marker genes, PD-L1, TGFB1, and IL10 were significantly correlated with FUCA2 expression in most tumor types. As we have known, there was a significant correlation between TGFB1 and IL-10 expression and TAMs. We observed that FUCA2 was significantly correlated with TGFB1 and IL-10 expression in most tumor types including, which may indicate the potential mechanism of FUCA2 influencing infiltration of TAMs.

### GSEA and GSVA Analysis of FUCA2 in Pan-Cancer

**Figure 9:**
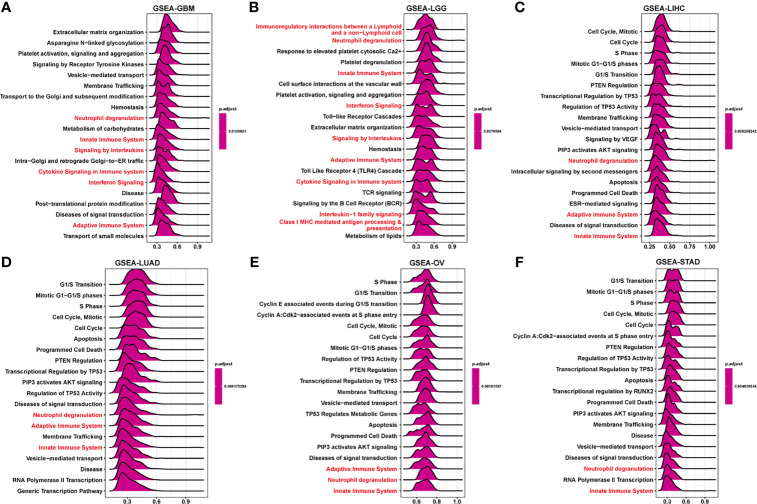
GSEA of FUCA2 in TCGA pan-cancer. **(A–F)** The top 20 significant pathways of FUCA2 GSEA results across the indicated tumor types. Red color represents immune-related pathways.

To elucidate the potential biological pathways regulated by FUCA2, we conducted GSEA using “clusterprofiler” in pan-cancer subjects, then selected the 12 tumors with similar results. Significantly enriched pathways presented positive NES and the results marked with red were the common occurrence across different tumors. These mainly focused on the mechanism of immune regulation, such as adaptive and innate immune systems, as well as neutrophil degranulation and cytokine signaling in the immune system (**Figure 9** and **Supplementary Figure 1**). Besides, cycle-related pathways (such as “Cell Cycle”, “Apoptosis”, and “AKT signaling”) are closely related to FUCA2 in pan-cancer. For GSVA results of 50 hallmark pathways from the MSigDB, we found that FUCA2 was associated with many cancer-promoting and immune-related pathway, including Glycolysis, PI3K AKT MTOR signaling, TGF BETA signaling, interferon alpha and interferon gamma response in pan-cancer (**Supplementary Figure 2**). Overall, these results indicated that FUCA2 plays a major role in tumor development and tumor immunity.

### FUCA2 Knockdown Inhibited the Cell Viability in Lung Cancer Cells

**Figure 10:**
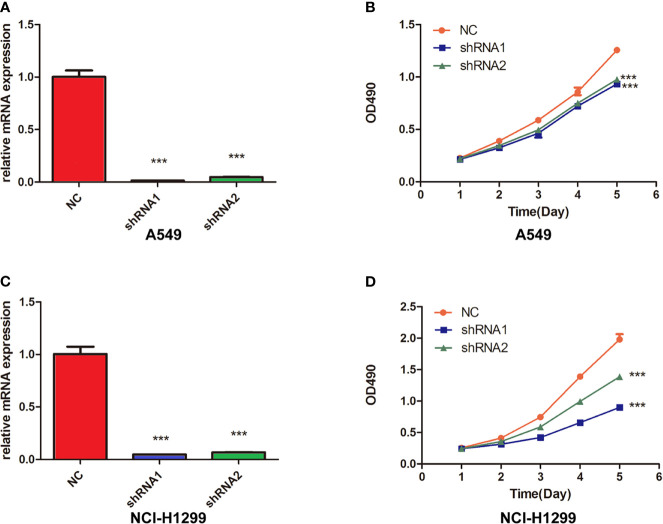
Knockdown of FUCA2 Inhibited the cell viability in Lung Cancer Cells. **(A)** A549 and cells were transfected with si-FUCA2, the level of FUCA2 was evaluated by qRT-PCR. **(B)** The cell viability of A549 cells was examined by MTT assay. **(C)** NCI-H1299 cells were transfected with si-FUCA2, the level of FUCA2 was evaluated by qRT-PCR. **(D)** The cell viability of NCI-H1299 cells was examined by MTT assay. ***P < 0.001.

To prove the oncogene role of FUCA2, we performed the experimental verification in lung cancer cells. We knocked down FUCA2 expression in A549 and NCI-H1299 cells *via* two FUCA2 shRNA, successfully (**Figures 10A, C**). Then, MTT assay showed that the inhibition of FUCA2 could significantly reduce the cell viability of lung cancer cell lines A549 (**Figure 10B**) and NCI-H1299 (**Figure 10D**), which was consistent with our conclusion.

## Discussion

Previous studies have shown that FUCA2 is a diagnostic marker in hepatocellular carcinoma and gastric cancer (11, 12). To date, however, little is known regarding its role in other cancer types. Results indicate that FUCA2 is overexpressed in 24 tumor types, relative to normal tissues, indicating that this gene mainly plays an oncogene role in tumor progression. Cox proportional hazards model analysis and Kaplan-Meier survival curves of prognostic value in pan-cancer revealed that its upregulation was markedly linked to worse OS of UVM, LUAD, LIHC, LGG, KICH, GBM, CESC, and MESO tumors. Further, FUCA2 expression was closely correlated with DNA methylation, and a high FUCA2 methylation level could serve as a biomarker of prognosis in patients with several cancers. These results suggested FUCA2 as a significant prognostic marker in indicated tumor types.

The TME, particularly the TIME, influence tumor development (16, 17). TME parameters can be exploited to assess response to tumor cell to immunotherapies, and has been also shown to influence clinical outcomes (18). Immune effector cells in the TME can suppress tumor growth (19). However, tumor cells may escape immune surveillance, through various mechanisms such as polarizing monocytes and macrophages towards an M2 phenotype, creating tumor associated macrophages (TAMs), thereby suppressing cytotoxicity of antitumor immune cells (20–22). Although some of the immune infiltration cells including TAMs, natural killer cells, B cells, and regulatory T cells have no inhibitory effects on cancer cells, they influence the ability of tumors to escape immune attack (5, 23). In the present study, we used three different methods to assess how FUCA2 levels correlate with infiltrating immune cells in pan-cancer, and obtained similar results. Specifically, FUCA2 levels showed a positive relationship with tumor infiltrating TAMs, especially M2-like macrophages, indicating that FUCA2 may contribute to macrophage polarization. FUCA2 may be a potential target to reduce the number of TAMs.

Our results further unraveled a strong link between FUCA2 level and immune-related genes, such as immunosuppressive genes, MHC genes, chemokines, and their receptors in most tumor types. Importantly, the positive correlations between FUCA2 expression and immunosuppressive genes, such as TGFB1, PD-L1, and IL10, further affirming the key role played by FUCA2 in regulation of tumor immunology, and macrophage polarization. TAMs, especially M2-like macrophages, which are particularly abundant in a tumor mass, contribute much to the immunosuppressive microenvironment (24). TGFB1 has been proved as the most predominant immune-suppressing molecular in the TME. TGFB1 can inhibit the generation, differentiation and function of effector T cells, as well as induce Tregs infiltration into TME (25). PD-L1 highly expressed on tumor cells was shown to promote apoptosis of antigen-specific and tumor-reactive T cells, leading to inhibition of anti-tumor immunity (26). Finally, our GSEA and GSVA results indicated that FUCA2 was linked to pathways involved in immune regulation in pan-cancer. These results indicated that FUCA2 level affects the TIME, and the high expression of FUCA2 indicated the immunosuppression microenvironment, providing a potential drug target for tumor immunotherapy.

To the best of our knowledge, this is the first pan-cancer analyses that focus on the value of FUCA2 in tumors. A comprehensive understanding of human cancers is important to identify specific targets or features for more precise and personalized treatment (27). Recently, pan-cancer analysis was used to discover common features and/or heterogeneities during vital biological processes that contribute to a dysregulation tumor microenvironment (28, 29). Therefore, pan-cancer analysis for identifying differential expressions and the role of FUCA2 in various tumors is clinical valuable. For example, We found that FUCA2 was over-expressed in most cancers and indicated a worse prognosis for several types of tumors, while FUCA2 was down-expressed and predicted a better prognosis for KIRC. The different results of survival analyses may provide guidance for clinical implications and theoretical basis for future studies in specific tumors. As we have found that FUCA2 has a potential cancer-promoting effect in pan-cancer, including LUAD, we further performed the experimental verification of FUCA2 in LUAD to prove its oncogene role. We found that the inhibition of FUCA2 could significantly reduce the proliferation rate of lung cancer cell lines A549 and NCI-H1299, which was consistent with our conclusion. We believe that these findings may be the foundation for prospective functional experiments and might eventually have an impact in the clinical setting.

However, considering that our study was mainly based on bioinformatics and rely on public databases, it had some major limitations. First, the patient data come entirely from open databases and have not been verified experimentally in clinic. Second, FUCA2 is highly expressed and is associated with poor prognosis in a variety of tumors, but the specific mechanism has not been verified. Therefore, further investigation should focus on clarifying the accuracy of an integrative analysis of FUCA2 expression and confirming the specific correlation of FUCA2 with tumor immunosuppressive microenvironment, respectively. Future more study to validate the expression and function of FUCA2 *in vivo* and *in vitro* is needed

In conclusion, our study suggested an oncogenic effect of FUCA2 and its potential as a prognostic biomarker in pan-cancer. High FUCA2 expression was predictive of high TAM infiltration and contributed to a tumor immunosuppressive microenvironment, providing a potential target for tumor therapy.

## Data Availability Statement

The datasets presented in this study can be found in online repositories. The names of the repository/repositories and accession number(s) can be found in the article/**Supplementary Material**.

## Ethics Statement

Ethical review and approval was not required for the study on human participants in accordance with the local legislation and institutional requirements. Written informed consent for participation was not required for this study in accordance with the national legislation and the institutional requirements.

## Author Contributions

AZ and MS designed the study. YX and TC collected the literature. AZ, TC, and XP analyzed the data. AZ and TC drafted the manuscript. MS modified the manuscript. All authors contributed to the article and approved the submitted version.

## Funding

This work was supported by grants from the Science Pre-research Foundation of the Second Affiliated Hospital of Soochow University (grant number SDFEYQN1909), Youth Science and Education Program of Suzhou (grant number KJXW2020018), Capacity improvement of respiratory clinical trial institutions in the Second Affiliated Hospital of Soochow University (grant number SLT201930) and Competitive discipline lift project of the Second Affiliated Hospital of Soochow University (grant number XKTJ-XK202007). 

## Conflict of Interest

The authors declare that the research was conducted in the absence of any commercial or financial relationships that could be construed as a potential conflict of interest.

## Publisher’s Note

All claims expressed in this article are solely those of the authors and do not necessarily represent those of their affiliated organizations, or those of the publisher, the editors and the reviewers. Any product that may be evaluated in this article, or claim that may be made by its manufacturer, is not guaranteed or endorsed by the publisher.

## Supplementary Material

Supplementary Figure 1 GSEA of FUCA2 in TCGA in several tumor types. **(A–F)** The top 20 significant pathways of FUCA2 GSEA results across the indicated tumor types. Red color represents immune-related pathways.

Supplementary Figure 2 GSVA of FUCA2. The heatmap shows the correlation between MFSD12 expression and GSVA scores of 50 Hallmark pathways in pan-cancer. *p < 0.05, **p < 0.01, ***p < 0.001, ****p < 0.0001.

Supplementary Table 1 The immune related genes which shows association with FUCA2.
