# Supplementary figures and images for: FUCA2 Is a Prognostic Biomarker and Correlated With an Immunosuppressive Microenvironment in Pan-Cancer
Source: Front Immunol. 2021 Oct 20;12:758648. doi: 10.3389/fimmu.2021.758648 (PMC8565374; doi:10.3389/fimmu.2021.758648)

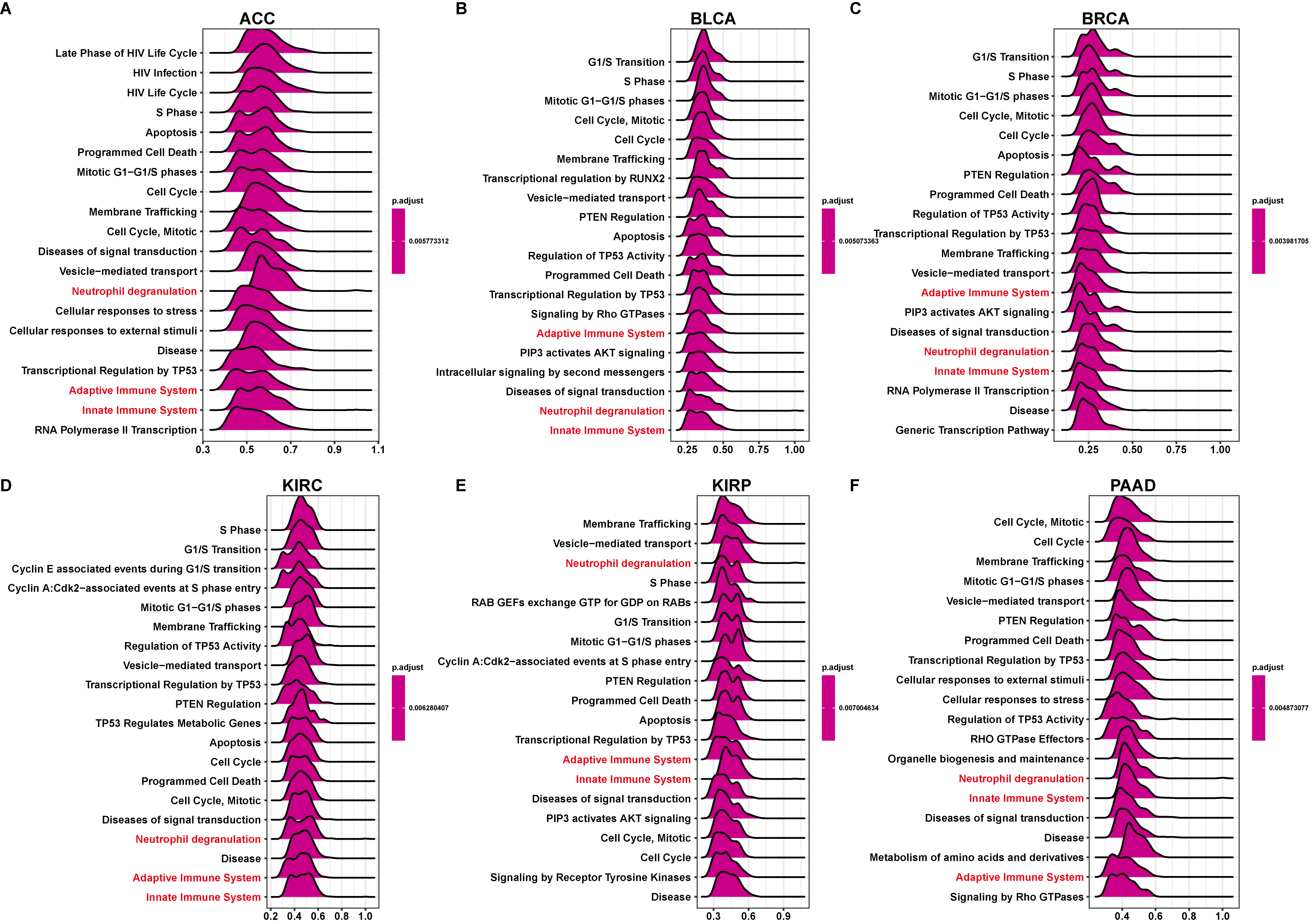

Supplement: Supplementary Figure 1 — GSEA of FUCA2 in TCGA in several tumor types. (A–F) The top 20 significant pathways of FUCA2 GSEA results across the indicated tumor types. Red color represents immune-related pathways. [file Image_1.tif]

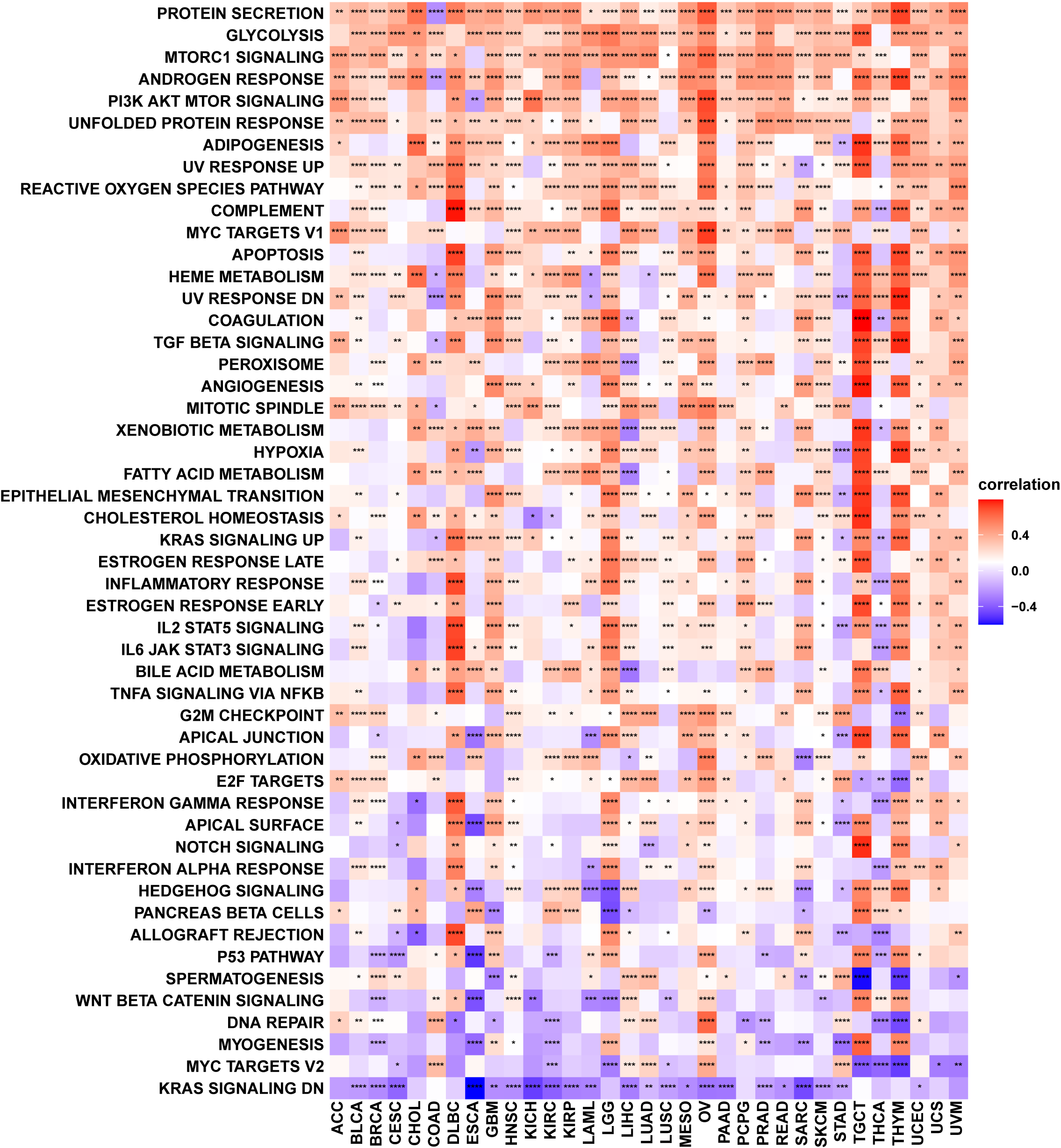

Supplement: Supplementary Figure 2 — GSVA of FUCA2. The heatmap shows the correlation between MFSD12 expression and GSVA scores of 50 Hallmark pathways in pan-cancer. *p < 0.05, **p < 0.01, ***p < 0.001, ****p < 0.0001. [file Image_2.tif]
